# How do breast cancer clinical trials approach cardiovascular safety: targeted or generalized?

**DOI:** 10.1186/s40959-024-00201-9

**Published:** 2024-02-07

**Authors:** Arsalan Hamid, Gregg C. Fonarow, Javed Butler, Michael E. Hall

**Affiliations:** 1 Department of Medicine, University of Mississippi Medical Center, Jackson, MS USA; 2 Division of Cardiology, David Geffen School of Medicine at UCLA, Los Angeles, CA USA; 3 Baylor Scott and White Research Institute, Dallas, TX USA; 4 Department of Medicine, Division of Cardiology, University of Mississippi Medical Center, Jackson, MS USA

**Keywords:** Breast cancer, Clinical trials, Cardiovascular safety, Prevent cardiotoxicity

## Abstract

**Background:**

Different breast cancer pharmacotherapy agents cause different forms of cardiovascular toxicity. We aim to assess if breast cancer pharmacotherapy trials approach cardiovascular safety in a targeted or generalized manner when administering different agents.

**Methods:**

We searched Embase and Medline for phase 2 and 3 breast cancer pharmacotherapy trials. We examined exclusion criterion for cardiovascular conditions and cardiovascular safety assessment through cardiovascular imaging, electrocardiogram, troponin, or natriuretic peptides. Fisher’s exact test was utilized to compare reporting.

**Results:**

Fifty breast cancer clinical trials were included in this study. Trials administering microtubule inhibitors were most likely to exclude patients with any CV condition compared with trials administering other agents (93.5% vs. 68.4%; *p* < 0.05), particularly coronary artery disease (77.4% vs. 36.8%; *p* < 0.01) but reported performing an electrocardiogram in 13 (41.9%) trials. Trials administering anti-HER 2 agents excluded all patients with at least one CV condition, particularly patients with heart failure (100.0% vs. 62.9%) and were more likely to perform echocardiograms (80.0% vs. 22.9%, *p* < 0.001) compared with other agents. Other agents excluded participants in a generalized manner and do not frequently perform targeted safety assessments.

**Conclusions:**

Only trials administering microtubule inhibitors or anti-HER 2 therapy exclude patients with cardiovascular disease in a targeted approach. However, anti-HER 2 therapy trials are the only breast cancer clinical trials that perform targeted safety assessments. Breast cancer clinical trials need to develop a targeted approach to cardiovascular safety assessments to permit inclusion of high-risk participants and generate clinical trial data generalizable to patients with cardiovascular disease undergoing cancer therapy.

**Supplementary Information:**

The online version contains supplementary material available at 10.1186/s40959-024-00201-9.

The advent of newer cancer therapies has led to improved survival in patients with cancer. However, cancer therapy–induced cardiovascular (CV) toxicity is a growing cause of morbidity and mortality in patients with cancer. Different agents are known to have various cellular targets and toxic CV effects [1]. Patients with clinical and subclinical CV disease (CVD) are frequently excluded from breast cancer trials and such trials do not assess CV safety [2]. Cancer therapy data needs to be representative of patients with CVD to make decisions in clinical practice. One approach to this is to selectively exclude high-risk patients and screen for toxicity specific to the agent. Thus, we assessed patterns of exclusion of CVD and safety assessment conduct amongst breast cancer pharmacotherapy trials, specific to the pharmacotherapy regimen administered.

The methods of our data collection have been previously published [2]. In brief we searched for cancer pharmacotherapy clinical trial articles that were: (1) phase 2 & 3 trials; (2) enrolled > 50 participants; and (3) assessed use of cancer pharmacotherapy. Data on reporting of exclusion due to CV conditions, CV safety assessments (electrocardiogram (ECG), cardiac imaging, troponin, and brain natriuretic peptide (BNP) levels), and CV adverse events were collected. Fisher’s exact test was applied to assess the association of reporting a CV exclusion or CV safety assessment with whether an agent was included or not included within a trial. A 2-sided *P* value of < 0.05 was considered significant.

In total, 1775 records were screened and 50 clinical trials that cumulatively enrolled 26,893 participants were included in our study. On average, trials had a population of 538 participants and a median age of 55.7 years. The most common agent administered was microtubule inhibitors (*n* = 31; 62% trials). Trials administering microtubule inhibitors were more likely to exclude patients with any CV condition compared with trials administering other agents (93.5% vs. 68.4%; *p* < 0.05; Table 1). Specifically, these trials were more likely to exclude patients with coronary artery disease (77.4% vs. 36.8%), hypertension (58.1% vs. 15.8%), and arrhythmias (71.0% vs. 36.8%) compared with other agents (*p* < 0.05 for all; Table 1). All trials administering anthracyclines and anti-HER 2 agents excluded patients with at least one CV condition. Heart failure (HF) was the most common exclusion criteria in trials administering anthracyclines (*n* = 13; 92.9%) and anti-HER 2 therapies (*n* = 15; 100%; Table 1).

**Table 1:** Cardiovascular exclusion and safety assessment reporting among various agents

	**Cardiovascular exclusion criteria, n (%)**							**Safety assessment, n (%)**		
	**Any CV exclusion**	**Heart failure/Low EF**	**Coronary artery disease**	**Hypertension**	**Arrhythmia**	**Pericardial disease**	**Valvular disease**	**Any CV safety assessment**	**Electrocardiogram**	**Cardiac imaging**
**Anthracycline included in regimen**										
**Yes**	14 (100.0)	13 (92.9)	11 (78.6)	10 (71.4)*	10 (71.4)	0 (0.0)	4 (28.6)	8 (57.1)	8 (57.1)	8 (57.1)
**No**	28 (77.8)	24 (66.7)	20 (55.6)	11 (30.6)	19 (52.8)	5 (13.9)	4 (11.1)	19 (52.8)	15 (41.7)	12 (33.3)
**Microtubule inhibitor included in regimen**										
**Yes**	29 (93.5)*	26 (83.9)	24 (77.4)†	18 (58.1)†	22 (71.0)*	3 (9.7)	7 (22.6)	16 (51.6)	13 (41.9)	13 (41.9)
**No**	13 (68.4)	11 (57.9)	7 (36.8)	3 (15.8)	7 (36.8)	2 (10.5)	1 (5.3)	11 (57.9)	10 (52.6)	7 (36.8)
**Alkylating agent included in regimen**										
**Yes**	20 (95.2)	17 (81.0)	15 (71.4)	11 (52.4)	14 (66.7)	0 (0.0)	6 (28.6)	11 (52.4)	10 (47.6)	10 (47.6)
**No**	22 (75.9)	20 (69.0)	16 (55.2)	10 (34.5)	15 (51.7)	5 (17.2)	2 (6.9)	16 (55.2)	13 (44.8)	10 (34.5)
**Kinase inhibitor included in regimen**										
**Yes**	9 (81.8)	9 (81.8)	6 (54.5)	4 (36.4)	6 (54.5)	0 (0.0)	2 (18.2)	8 (72.7)	7 (63.6)	6 (54.5)
**No**	33 (84.6)	28 (71.8)	25 (64.1)	17 (43.6)	23 (59.0)	5 (12.8)	6 (15.4)	19 (48.7)	16 (41.0)	14 (35.9)
**Antibody to HER-2 included in regimen**										
**Yes**	15 (100.0)	15 (100.0)†	10 (66.7)	7 (46.7)	9 (60.0)	1 (6.7)	5 (33.3)*	12 (80.0)*	10 (66.7)	12 (80.0)‡
**No**	27 (77.1)	22 (62.9)	21 (60.0)	14 (40.0)	20 (57.1)	4 (11.4)	3 (8.6)	15 (42.9)	13 (37.1)	8 (22.9)
**Aromatase inhibitor included in regimen**										
**Yes**	8 (61.5)	7 (53.8)	4 (30.8)	2 (15.4)	4 (30.8)	2 (15.4)	0 (0.0)	6 (46.2)	5 (38.5)	4 (30.8)
**No**	34 (91.9)*	30 (81.1)	27 (73.0)*	19 (51.4)*	25 (67.6)*	3 (8.1)	8 (21.6)	21 (56.8)	18 (48.6)	16 (43.2)
**Estrogen receptor binder included in regimen**										
**Yes**	6 (85.7)	5 (71.4)	3 (42.9)	2 (28.6)	3 (42.9)	3 (42.9)*	0 (0.0)	5 (71.4)	3 (42.9)	3 (42.9)
**No**	36 (83.7)	32 (74.4)	28 (65.1)	19 (44.2)	26 (60.5)	2 (4.7)	8 (18.6)	22 (51.2)	20 (46.5)	17 (39.5)
**Antimetabolite included in regimen**										
**Yes**	10 (83.3)	10 (83.3)	7 (58.3)	5 (41.7)	6 (50.0)	0 (0.0)	2 (16.7)	7 (58.3)	7 (58.3)	5 (41.7)
**No**	32 (84.2)	27 (71.1)	24 (63.2)	16 (42.1)	23 (60.5)	5 (13.2)	6 (15.8)	20 (52.6)	16 (42.1)	15 (39.5)
**Platinum included in regimen**										
**Yes**	6 (85.7)	5 (71.4)	4 (57.1)	4 (57.1)	4 (57.1)	0 (0.0)	2 (28.6)	4 (57.1)	3 (42.9)	4 (57.1)
**No**	36 (83.7)	32 (74.4)	27 (62.8)	17 (39.5)	25 (58.1)	5 (11.6)	6 (14.0)	23 (53.5)	20 (46.5)	16 (37.2)

*CV* Cardiovascular, *EF* Ejection fraction

^*^Indicates *p*-value <0.05

^†^Indicates *p*-value <0.01

^‡^Indicates *p*-value <0.001

Trials administering anti-HER 2 agents were more likely to conduct CV safety assessments compared with other agents (80.0% vs. 42.9%; *p* < 0.05). The most common safety assessment reported in these trials was echocardiography and they were more likely to perform echocardiography than trials administering other agents (80.0% vs. 22.9%; *p* < 0.001). However, there was no significant difference in the conduct of ECG compared with other agents (Table 1). Lastly, no BNP or troponin collection was reported in any trial irrespective of the agent administered.

While it is known that breast cancer trials frequently exclude patients with prevalent CV conditions, individual assessment amongst different agents has never been reported [2, 3]. In this study we highlight that breast cancer trials administering some agents have a targeted approach to excluding patients at high-risk, such as the risk of HF from anti-HER 2 agents or the risk of cardiac ischemia and arrhythmias from microtubule inhibitors [1]. Nonetheless, other agents such as alkylating agents, kinase inhibitors, estrogen receptor binders, antimetabolites, and platinum containing regimens do not seem to have a targeted approach compared to other agents and may exclude patients with CV conditions in a generalized manner.

Breast cancer trials administering microtubule inhibitors may be aware of the risk of cardiac ischemia or arrhythmias as such trials selectively exclude such patients, but only report performing an ECG in 13 (41.9%) trials. Similarly, patients with HF are excluded from breast cancer trials administering anthracyclines as they are known to be at risk but only 8 (57.1%) breast cancer trials administering anthracyclines report performing echocardiography. Exclusion of high-risk patients may be supported by trialists in favor of generating trial results with the primary outcome in focus. However, absence of targeted screening to identify potential CV toxicity is not justifiable as it is an achievable measure that will not impact trial results. Anti-HER 2 breast cancer trials are an example where patients with HF are selectively excluded but these trials are also more likely to perform echocardiography compared with other agents (*p* < 0.001).

The recent European Society of Cardiology (ESC) 2022 guidelines on cardio-oncology recommend the use of risk stratifying tools [4]. Only patients meeting high or very high-risk criteria require discussions of the risk/benefit balance of cardiotoxic anticancer treatment rather than direct exclusion. Furthermore, all patients undergoing therapy with anthracyclines and anti-HER 2 therapy require an echocardiogram (Class 1B recommendation), and biomarker assessment (BNP or high sensitivity cardiac troponin-T) can be considered in low/moderate risk patients (Class 2 recommendation) [4]. Patients of breast cancer clinical trials have thus been excluded from treatment which they otherwise may receive and then do not receive screening for the CV toxicity the agent is at risk of inducing.

Agents such as anthracyclines have had an abundance of clinical trials. While they are known to be associated with HF and CV dysfunction, they are not particularly known to cause coronary artery disease. We observed that in over three-quarters of occasions where an anthracycline is included in the trial regimen, the trial will exclude patients with coronary artery disease. This may be due to another agent included in the regimen or due to the future risk of HF with ischemic cardiomyopathy. However, biomarker-based risk stratification, frequent echocardiographic follow-up, and most importantly medical optimization with statins, beta-blockers, angiotensin receptor antagonists, and dexrazoxane can be utilized to prevent LVEF decline and incident HF [5]. In the future, initiation or continuation of such medical therapy at enrollment in such patients can be a pathway by which cancer trials can include higher risk patients while minimizing their risk of future events. Several of these medications are likely to have a minimal impact on the conduct of the trial or interpretation of results but would need assessment on a case-to-case basis to evaluate if the trial therapy itself interacts with a certain cardioprotective agent. By frequent use of such cardioprotective therapies in cancer trials, higher risk populations can safely be included and thus increase the generalizability of trial data. However, newer agents such as tyrosine kinase inhibitors initially require a more nuanced approach. Initially trials may consider a narrow inclusion population with judicious conduct of all-inclusive CV safety assessments with frequent biomarker, imaging, and ECG assessment to favor identifying conditions to considered adverse effects of the therapy. Subsequent trials are then needed to identify therapeutic and preventive options for cardiotoxicity from such cancer therapies. Once data accumulates on a therapy, more inclusive trials should then be conducted to generate generalizable data for higher risk individuals, and only exclusions pertinent to high-risk common adverse conditions should be implemented. In the example of tyrosine kinase inhibitors, they cause a wide range of CV conditions ranging from hypertension to HF [6]. Conditions such as hypertension are highly prevalent but not particularly high-risk, and can be treated as well as managed during the trial and so do not require exclusion, but are occasionally still excluded (*n* = 4; 36.4% trials) in contemporary trials (Table 1).

In the future, cancer trials assessing agents with some prior safety data should be more inclusive of CV conditions the agent is not particularly high-risk for. Targeted exclusion of high-risk CV conditions with high event rates in prior literature may be considered bounds for exclusion of a CV condition, but otherwise trials should be more inclusive of CV conditions to generate data generalizable to patients with CV conditions. Furthermore, future cancer trials should aim to conduct CV safety assessments more judiciously for subclinical and clinical CVD the agent is known to cause as well as that which it is not known to cause to detect subclinical disease missed in prior trials as well as permit trials to include higher risk individuals by close monitoring.

In conclusion, breast cancer pharmacotherapy trials administering anti-HER 2 therapies and microtubule inhibitors have a targeted approach to excluding high-risk patients while trials administering other agents do not. Furthermore, only trials administering anti-HER 2 therapies have modest targeted CV screening but trials administering other agents neither have a targeted approach to screening, nor an extensive one. In the future, Initial risk stratification with screening and subsequently targeted exclusion (of patients at very high-risk) or closer monitoring with improved targeted CV screening assessments pertinent to the agent administered is needed. This would permit a pragmatic approach to generating later phase clinical trial data generalizable to patients with CVD undergoing cancer therapy.

### Supplementary Information

**Additional file 1: Supplemental Table 1. **List of all included studies.

## Data Availability

No datasets were generated or analysed during the current study.

## Abbreviations

BNP: Brain Natriuretic Peptide
CV: Cardiovascular
CVD: Cardiovascular Disease
ECG: Electrocardiogram
ESC: European Society of Cardiology
HF: Heart failure
