# Supplementary material for: How do breast cancer clinical trials approach cardiovascular safety: targeted or generalized?
Source: Cardiooncology. 2024 Feb 7;10:6. doi: 10.1186/s40959-024-00201-9 (PMC10848621; doi:10.1186/s40959-024-00201-9)
Supplement: Supplementary file 1 — Additional file 1: Supplemental Table 1. List of all included studies. [file 40959_2024_201_MOESM1_ESM.docx]

| **Supplemental Table 1. List of all included studies** | | |
| --- | --- | --- |
| **Serial Number** | **Title** | **Citation** |
| 1 | Sorafenib in combination with docetaxel as first-line therapy for HER2-negative metastatic breast cancer: Final results of the randomized, double-blind, placebo-controlled phase II MADONNA study | Mavratzas A, Baek S, Gerber B, Schmidt M, Moebus V, Foerster F, Grischke EM, Fasching P, Strumberg D, Solomayer E, et al. Sorafenib in combination with docetaxel as first-line therapy for HER2-negative metastatic breast cancer: Final results of the randomized, double-blind, placebo-controlled phase II MADONNA study. *Breast*. 2019;45:22-28 |
| 2 | Randomized phase II study evaluating weekly oral vinorelbine versus weekly paclitaxel in estrogen receptor-positive, HER2-negative patients with advanced breast cancer (NorBreast-231 trial) | Aapro M, Ruiz-Borrego M, Hegg R, Kukielka-Budny B, Morales S, Cinieri S, Freitas-Junior R, Garcia-Estevez L, Szombara E, Borges GS, et al. Randomized phase ii study evaluating weekly oral vinorelbine versus weekly paclitaxel in estrogen receptor-positive, HER2-negative patients with advanced breast cancer (NorBreast-231 trial). *Breast*. 2019;45:7-14 |
| 3 | Concurrent neoadjuvant chemotherapy and estrogen deprivation in patients with estrogen receptor‐positive, human epidermal growth factor receptor 2‐negative breast cancer (CBCSG‐036): A randomized, controlled, multicenter trial | Yu KD, Wu SY, Liu GY, Wu J, Di GH, Hu Z, Hou YF, Chen CM, Fan L, Tang LC, et al. Concurrent neoadjuvant chemotherapy and estrogen deprivation in patients with estrogen receptor-positive, human epidermal growth factor receptor 2-negative breast cancer (CBCSG‐036): A randomized, controlled, multicenter trial. *Cancer*. 2019;125:2185-2193 |
| 4 | A multi-national, randomised, open-label, parallel, phase III non-inferiority study comparing NK105 and paclitaxel in metastatic or recurrent breast cancer patients | Fujiwara Y, Mukai H, Saeki T, Ro J, Lin YC, Nagai SE, Lee KS, Watanabe J, Ohtani S, Kim SB, et al. A multi-national, randomised, open-label, parallel, phase III non-inferiority study comparing NK105 and paclitaxel in metastatic or recurrent breast cancer patients. *Br J Cancer*. 2019;120:475-480 |
| 5 | Efficacy and safety of everolimus plus exemestane in postmenopausal women with hormone receptor‐positive, human epidermal growth factor receptor 2‐negative locally advanced or metastatic breast cancer: Results of the single‐arm, phase IIIB 4EVER trial | Tesch H, Stoetzer O, Decker T, Kurbacher CM, Marme F, Schneeweiss A, Mundhenke C, Distelrath A, Fasching PA, Lux MP, et al. Efficacy and safety of everolimus plus exemestane in postmenopausal women with hormone receptor-positive, human epidermal growth factor receptor 2-negative locally advanced or metastatic breast cancer: Results of the single-arm, phase IIIB 4EVER trial. *Int J Cancer*. 2019;144:877-885 |
| 6 | PF-05280014 (a trastuzumab biosimilar) plus paclitaxel compared with reference trastuzumab plus paclitaxel for HER2-positive metastatic breast cancer: a randomised, double-blind study | Pegram MD, Bondarenko I, Zorzetto MMC, Hingmire S, Iwase H, Krivorotko PV, Lee KS, Li RK, Pikiel J, Aggarwal R, et al. PF-05280014 (a trastuzumab biosimilar) plus paclitaxel compared with reference trastuzumab plus paclitaxel for HER2-positive metastatic breast cancer: A randomised, double-blind study. *Br J Cancer*. 2019;120:172-182 |
| 7 | Randomized Phase II Study Evaluating Palbociclib in Addition to Letrozole as Neoadjuvant Therapy in Estrogen Receptor–Positive Early Breast Cancer: PALLET Trial | Johnston S, Puhalla S, Wheatley D, Ring A, Barry P, Holcombe C, Boileau JF, Provencher L, Robidoux A, Rimawi M, et al. Randomized phase II study evaluating palbociclib in addition to letrozole as neoadjuvant therapy in estrogen receptor-positive early breast cancer: PALLET Trial. *J Clin Oncol*. 2019;37:178-189 |
| 8 | Safety, activity, and molecular heterogeneity following neoadjuvant non-pegylated liposomal doxorubicin, paclitaxel, trastuzumab, and pertuzumab in HER2-positive breast cancer (Opti-HER HEART): an open-label, single-group, multicenter, phase 2 trial | Gavila J, Oliveira M, Pascual T, Perez-Garcia J, Gonzalez X, Canes J, Pare L, Calvo I, Ciruelos E, Munoz M, et al. Safety, activity, and molecular heterogeneity following neoadjuvant non-pegylated liposomal doxorubicin, paclitaxel, trastuzumab, and pertuzumab in HER2-positive breast cancer (Opti-HER HEART): An open-label, single-group, multicenter, phase 2 trial. *BMC Med*. 2019;17:8 |
| 9 | Intense dose-dense epirubicin, paclitaxel, cyclophosphamide versus weekly paclitaxel, liposomal doxorubicin (plus carboplatin in triple-negative breast cancer) for neoadjuvant treatment of high-risk early breast cancer (GeparOcto—GBG 84): A randomised phase III trial | Schneeweiss A, Mobus V, Tesch H, Hanusch C, Denkert C, Lubbe K, Huober J, Klare P, Kummel S, Untch M, et al. Intense dose-dense epirubicin, paclitaxel, cyclophosphamide versus weekly paclitaxel, liposomal doxorubicin (plus carboplatin in triple-negative breast cancer) for neoadjuvant treatment of high-risk early breast cancer (GeparOcto—GBG 84): A randomised phase III trial. *Eur J Cancer*. 2019;106:181-192 |
| 10 | A randomized and open-label phase II trial reports the efficacy of neoadjuvant lobaplatin in breast cancer | Wu X, Tang P, Li S, Wang S, Liang Y, Zhong L, Ren L, Zhang T, Zhang Y. A randomized and open-label phase ii trial reports the efficacy of neoadjuvant lobaplatin in breast cancer. *Nat Commun*. 2018;9:832 |
| 11 | Neoadjuvant chemotherapy with or without anthracyclines in the presence of dual HER2 blockade for HER2-positive breast cancer (TRAIN-2): a multicentre, open-label, randomised, phase 3 trial | van Ramshorst MS, van der Voort A, van Werkhoven ED, Mandjes IA, Kemper I, Dezentje VO, Oving IM, Honkoop AH, Tick LW, van de Wouw AJ, et al. Neoadjuvant chemotherapy with or without anthracyclines in the presence of dual her2 blockade for HER2-positive breast cancer (TRAIN-2): A multicentre, open-label, randomised, phase 3 trial. *Lancet Oncol*. 2018;19:1630-1640 |
| 12 | A Randomized Phase II Study of Eribulin/Cyclophosphamide or Docetaxel/Cyclophosphamide as Neoadjuvant Therapy in Operable HER2-negative Breast Cancer | Yardley DA, Shipley D, Zubkus J, Wright GL, Ward PJ, Mani A, Shastry M, Finney L, DeBusk L, Hainsworth JD. A randomized phase ii study of eribulin/cyclophosphamide or docetaxel/cyclophosphamide as neoadjuvant therapy in operable HER2-negative breast cancer. *Clin Breast Cancer*. 2019;19:1-9 |
| 13 | Atezolizumab and nab-paclitaxel in advanced triple-negative breast cancer | Schmid P, Adams S, Rugo HS, Schneeweiss A, Barrios CH, Iwata H, Dieras V, Hegg R, Im SA, Shaw Wright G, et al. Atezolizumab and nab-paclitaxel in advanced triple-negative breast cancer. *N Engl J Med*. 2018;379:2108-2121 |
| 14 | Combination versus sequential paclitaxel plus gemcitabine as first-line chemotherapy for women with metastatic breast cancer: a prospective randomized phase II study. | Shao B, Song G, Li H, Dil L, Jiang H, Liang X, Yan Y, Zhang R, Ran R, Wang J, el al. Combination versus sequential paclitaxel plus gemcitabine as first-line chemotherapy for women with metastatic breast cancer: A prospective randomized phase II study. *J BUON*. 2018;23:1583-1590 |
| 15 | Fulvestrant plus goserelin versus anastrozole plus goserelin versus goserelin alone for hormone receptor-positive, HER2-negative tamoxifen-pretreated premenopausal women with recurrent or metastatic breast cancer (KCSG BR10-04): a multicentre, open-label, three-arm, randomised phase II trial (FLAG study) | Kim JY, Im SA, Jung KH, Ro J, Sohn J, Kim JH, Park YH, Kim TY, Kim SB, Lee KS, et al. Fulvestrant plus goserelin versus anastrozole plus goserelin versus goserelin alone for hormone receptor-positive, her2-negative tamoxifen-pretreated premenopausal women with recurrent or metastatic breast cancer (KCSG BR10-04): A multicentre, open-label, three-arm, randomised phase II trial (FLAG study). *Eur J Cancer*. 2018;103:127-136 |
| 16 | Intermittent versus continuous first-line treatment for HER2-negative metastatic breast cancer: the Stop & Go study of the Dutch Breast Cancer Research Group (BOOG) | Claessens AKM, Bos M, Lopez-Yurda M, Bouma JM, Rademaker-Lakhai JM, Honkoop AH, de Graaf H, van Druten E, van Warmerdam LJC, van der Sangen MJC, et al. Intermittent versus continuous first-line treatment for her2-negative metastatic breast cancer: The stop & go study of the dutch breast cancer research group (BOOG). *Breast Cancer Res Treat*. 2018;172:413-423 |
| 17 | Paclitaxel With Inhibitor of Apoptosis Antagonist, LCL161, for Localized Triple-Negative Breast Cancer, Prospectively Stratified by Gene Signature in a Biomarker-Driven Neoadjuvant Trial | Bardia A, Parton M, Kummel S, Estevez LG, Huang CS, Cortes J, Ruiz-Borrego M, Telli ML, Martin-Martorell P, Lopez R, et al. Paclitaxel with inhibitor of apoptosis antagonist, LCL161, for localized triple-negative breast cancer, prospectively stratified by gene signature in a biomarker-driven neoadjuvant trial. *J Clin Oncol*. 2018:JCO2017748392 |
| 18 | Multicenter Phase II Study of Lurbinectedin in BRCA-Mutated and Unselected Metastatic Advanced Breast Cancer and Biomarker Assessment Substudy | Cruz C, Llop-Guevara A, Garber JE, Arun BK, Perez Fidalgo JA, Lluch A, Telli ML, Fernandez C, Kahatt C, Galmarini CM, et al. Multicenter phase II study of lurbinectedin in BRCA -mutated and unselected metastatic advanced breast cancer and biomarker assessment substudy. *J Clin Oncol*. 2018;36:3134-3143 |
| 19 | Everolimus Plus Exemestane vs Everolimus or Capecitabine Monotherapy for Estrogen Receptor–Positive, HER2-Negative Advanced Breast CancerThe BOLERO-6 Randomized Clinical Trial | Jerusalem G, de Boer RH, Hurvitz S, Yardley DA, Kovalenko E, Ejlertsen B, Blau S, Ozguroglu M, Landherr L, Ewertz M, et al. Everolimus plus exemestane vs everolimus or capecitabine monotherapy for estrogen receptor-positive, HER2-negative advanced breast cancer: The BOLERO-6 randomized clinical trial. *JAMA Oncol*. 2018;4:1367-1374 |
| 20 | UCBG 2-08: 5-year efficacy results from the UNICANCER-PACS08 randomised phase III trial of adjuvant treatment with FEC100 and then either docetaxel or ixabepilone in patients with early-stage, poor prognosis breast cancer | Campone M, Lacroix-Triki M, Roca L, Spielmann M, Wildiers H, Cottu P, Kerbrat P, Levy C, Desmoulins I, Bachelot T, et al. UCBG 2-08: 5-year efficacy results from the UNICANCER-PACS08 randomised phase iii trial of adjuvant treatment with FEC100 and then either docetaxel or ixabepilone in patients with early-stage, poor prognosis breast cancer. *Eur J Cancer*. 2018;103:184-194 |
| 21 | Adjuvant dose-dense doxorubicin-cyclophosphamide versus docetaxel-doxorubicin-cyclophosphamide for high-risk breast cancer: First results of the randomised MATADOR trial (BOOG 2004-04) | van Rossum AGJ, Kok M, van Werkhoven E, Opdam M, Mandjes IAM, van Leeuwen-Stok AE, van Tinteren H, Imholz ALT, Portielje JEA, et al. Adjuvant dose-dense doxorubicin-cyclophosphamide versus docetaxel-doxorubicin-cyclophosphamide for high-risk breast cancer: First results of the randomised MATADOR trial (BOOG 2004-04). *Eur J Cancer*. 2018;102:40-48 |
| 22 | Results From the First Multicenter, Open-label, Phase IIIb Study Investigating the Combination of Pertuzumab With Subcutaneous Trastuzumab and a Taxane in Patients With HER2-positive Metastatic Breast Cancer (SAPPHIRE) | Woodward N, De Boer RH, Redfern A, White M, Young J, Truman M, Beith J. Results from the first multicenter, open-label, phase IIIb study investigating the combination of pertuzumab with subcutaneous trastuzumab and a taxane in patients with HER2-positive metastatic breast cancer (SAPPHIRE). *Clin Breast Cancer*. 2019;19:216-224 |
| 23 | Effect of Adjuvant Trastuzumab for a Duration of 9 Weeks vs 1 Year With Concomitant Chemotherapy for Early Human Epidermal Growth Factor Receptor 2–Positive Breast Cancer: The SOLD Randomized Clinical Trial | Joensuu H, Fraser J, Wildiers H, Huovinen R, Auvinen P, Utriainen M, Nyandoto P, Villman KK, Halonen P, Granstam-Bjorneklett H, et al. Effect of adjuvant trastuzumab for a duration of 9 weeks vs 1 year with concomitant chemotherapy for early human epidermal growth factor receptor 2-positive breast cancer: The SOLD randomized clinical trial. *JAMA Oncol.* 2018;4:1199-1206 |
| 24 | Double-Blind Phase III Trial of Adjuvant Chemotherapy With and Without Bevacizumab in Patients With Lymph Node–Positive and High-Risk Lymph Node–Negative Breast Cancer (E5103) | Miller KD, O'Neill A, Gradishar W, Hobday TJ, Goldstein LJ, Mayer IA, Bloom S, Brufsky AM, Tevaarwerk AJ, Sparano JA, et al. Double-blind phase III trial of adjuvant chemotherapy with and without bevacizumab in patients with lymph node-positive and high-risk lymph node-negative breast cancer (E5103). *J Clin Oncol*. 2018;36:2621-2629 |
| 25 | Talazoparib in patients with advanced breast cancer and a germline BRCA mutation | Litton JK, Rugo HS, Ettl J, Hurvitz SA, Goncalves A, Lee KH, Fehrenbacher L, Yerushalmi R, Mina LA, Martin M, et al. Talazoparib in patients with advanced breast cancer and a germline brca mutation. *N Engl J Med*. 2018;379:753-763 |
| 26 | Phase III Randomized Study of Ribociclib and Fulvestrant in Hormone Receptor–Positive, Human Epidermal Growth Factor Receptor 2–Negative Advanced Breast Cancer: MONALEESA-3 | Slamon DJ, Neven P, Chia S, Fasching PA, De Laurentiis M, Im SA, Petrakova K, Bianchi GV, Esteva FJ, Martin M, et al. Phase iii randomized study of ribociclib and fulvestrant in hormone receptor-positive, human epidermal growth factor receptor 2-negative advanced breast cancer: MONALEESA-3. *J Clin Oncol*. 2018;36:2465-2472 |
| 27 | A randomized, double-blind, phase 2 study of ruxolitinib or placebo in combination with capecitabine in patients with advanced HER2-negative breast cancer and elevated C-reactive protein, a marker of systemic inflammation | O'Shaughnessy J, DeMichele A, Ma CX, Richards P, Yardley DA, Wright GS, Kalinsky K, Steis R, Diab S, Kennealey G, et al. A randomized, double-blind, phase 2 study of ruxolitinib or placebo in combination with capecitabine in patients with advanced her2-negative breast cancer and elevated c-reactive protein, a marker of systemic inflammation. *Breast Cancer Res Treat*. 2018;170:547-557 |
| 28 | A randomized phase II trial of trastuzumab plus capecitabine versus lapatinib plus capecitabine in patients with HER2-positive metastatic breast cancer previously treated with trastuzumab and taxanes: WJOG6110B/ELTOP | Takano T, Tsurutani J, Takahashi M, Yamanaka T, Sakai K, Ito Y, Fukuoka J, Kimura H, Kawabata H, Tamura K, et al. A randomized phase ii trial of trastuzumab plus capecitabine versus lapatinib plus capecitabine in patients with her2-positive metastatic breast cancer previously treated with trastuzumab and taxanes: WJOB6110B/ELTOP*. Breast.* 2018;40:67-75 |
| 29 | Neoadjuvant PF-05280014 (a potential trastuzumab biosimilar) versus trastuzumab for operable HER2+ breast cancer | Lammers PE, Dank M, Masetti R, Abbas R, Hilton F, Coppola J, Jacobs I. Neoadjuvant PF-05280014 (a potential trastuzumab biosimilar) versus trastuzumab for operable HER2+ breast cancer. *Br J Cancer.* 2018;119:266-273 |
| 30 | Efficacy and safety of ABP 980 compared with reference trastuzumab in women with HER2-positive early breast cancer (LILAC study): a randomised, double-blind, phase 3 trial | von Minckwitz G, Colleoni M, Kolberg HC, Morales S, Santi P, Tomasevic Z, Zhang N, Hanes V. Efficacy and safety of ABP 980 compared with reference trastuzumab in women with HER2-positive early breast cancer (LILAC study): A randomised, double-blind, phase 3 trial. *Lancet Oncol*. 2018;19:987-998 |
| 31 | Bi-weekly eribulin therapy for metastatic breast cancer: a multicenter phase II prospective study (JUST-STUDY) | Ohtani S, Nakayama T, Yoshinami T, Watanabe KI, Hara F, Sagara Y, Kawaguchi H, Higaki K, Matsunami N, Hasegawa Y, et al. Bi-weekly eribulin therapy for metastatic breast cancer: A multicenter phase II prospective study (JUST-STUDY). *Breast Cancer*. 2018;25:438-446 |
| 32 | Efficacy and safety of trastuzumab, lapatinib, and paclitaxel neoadjuvant treatment with or without prolonged exposure to anti-HER2 therapy, and with or without hormone therapy for HER2-positive primary breast cancer: a randomised, five-arm, multicentre, open-label phase II trial | Masuda N, Toi M, Yamamoto N, Iwata H, Kuroi K, Bando H, Ohtani S, Takano T, Inoue K, Yanagita Y, et al. Efficacy and safety of trastuzumab, lapatinib, and paclitaxel neoadjuvant treatment with or without prolonged exposure to anti- HER2 therapy, and with or without hormone therapy for HER2-positive primary breast cancer: A randomised, five-arm, multicentre, open-label phase II trial. *Breast Cancer*. 2018;25:407-415 |
| 33 | Ribociclib plus endocrine therapy for premenopausal women with hormone-receptor-positive, advanced breast cancer (MONALEESA-7): a randomised phase 3 trial | Tripathy D, Im SA, Colleoni M, Franke F, Bardia A, Harbeck N, Hurvitz SA, Chow L, Sohn J, Lee KS, et al. Ribociclib plus endocrine therapy for premenopausal women with hormone-receptor-positive, advanced breast cancer (MONALEESA-7): A randomised phase 3 trial*. Lancet Oncol*. 2018;19:904-915 |
| 34 | Neoadjuvant endocrine therapy with exemestane followed by response‐guided combination therapy with low‐dose cyclophosphamide in postmenopausal patients with estrogen receptor‐positive breast cancer: A multicenter, open‐label, phase II study | Sato N, Masuda N, Morimoto T, Ueno T, Kanbayashi C, Kaneko K, Yasojima H, Saji S, Sasano H, Morita S, et al. Neoadjuvant endocrine therapy with exemestane followed by response-guided combination therapy with low-dose cyclophosphamide in postmenopausal patients with estrogen receptor-positive breast cancer: A multicenter, open-label, phase II study. *Cancer Med*. 2018 |
| 35 | Induction therapy with paclitaxel and bevacizumab followed by switch maintenance therapy with eribulin in Japanese patients with HER2-negative metastatic breast cancer: A multicenter, collaborative, open-label, phase II clinical study for the SBCCSG 35 investigators | Inoue K, Ninomiya J, Saito T, Kimizuka K, Kurosumi M. Induction therapy with paclitaxel and bevacizumab followed by switch maintenance therapy with eribulin in japanese patients with HER2-negative metastatic breast cancer: A multicenter, collaborative, open-label, phase II clinical study for the SBCCSG 35 investigators. *BMC Cancer*. 2018;18:671 |
| 36 | The efficacy and feasibility of dose-dense sequential chemotherapy for Japanese patients with breast cancer | Takabatake D, Kajiwara Y, Ohtani S, Itano Y, Yamamoto M, Kubo S, Ikeda M, Takahashi M, Hara F, Aogi K, et al. The efficacy and feasibility of dose-dense sequential chemotherapy for japanese patients with breast cancer. *Breast Cancer*. 2018;25:717-722 |
| 37 | Randomized phase II trial of fulvestrant plus everolimus or placebo in postmenopausal women with hormone receptor-positive, human epidermal growth factor receptor 2-negative metastatic breast cancer resistant to aromatase inhibitor therapy: Results of PrE0102 | Kornblum N, Zhao F, Manola J, Klein P, Ramaswamy B, Brufsky A, Stella PJ, Burnette B, Telli M, Makower DF, et al. Randomized phase ii trial of fulvestrant plus everolimus or placebo in postmenopausal women with hormone receptor-positive, human epidermal growth factor receptor 2-negative metastatic breast cancer resistant to aromatase inhibitor therapy: Results of PrE0102. *J Clin Oncol*. 2018;36:1556-1563 |
| 38 | A randomized Phase III trial of neoadjuvant recombinant human endostatin, docetaxel and epirubicin as first-line therapy for patients with breast cancer (CBCRT01) | Chen J, Yao Q, Huang M, Wang B, Zhang J, Wang T, Ming Y, Zhou X, Jia Q, Huan Y, et al. A randomized phase iii trial of neoadjuvant recombinant human endostatin, docetaxel and epirubicin as first-line therapy for patients with breast cancer (CBCRT01). *Int J Cancer.* 2018;142:2130-2138 |
| 39 | Carboplatin in BRCA1/2-mutated and triple-negative breast cancer BRCAness subgroups: The TNT Trial | Tutt A, Tovey H, Cheang MCU, Kernaghan S, Kilburn L, Gazinska P, Owen J, Abraham J, Barrett S, Barrett-Lee P, et al. Carboplatin in brca1/2-mutated and triple-negative breast cancer BRCAness subgroups: The TNT Trial. *Nat Med*. 2018;24:628-637 |
| 40 | Open-label randomised phase III trial of vinflunine versus an alkylating agent in patients with heavily pretreated metastatic breast cancer | Cortes J, Perez-Garcia J, Levy C, Gomez Pardo P, Bourgeois H, Spazzapan S, Martinez-Janez N, Chao TC, Espie M, Nabholtz JM, et al. Open-label randomised phase III trial of vinflunine versus an alkylating agent in patients with heavily pretreated metastatic breast cancer. *Ann Oncol*. 2018;29:881-887 |
| 41 | Everolimus Plus Letrozole for Treatment of Patients With HR(+), HER2(–) Advanced Breast Cancer Progressing on Endocrine Therapy: An Open-label, Phase II Trial | Safra T, Kaufman B, Kadouri L, Efrat Ben-Baruch N, Ryvo L, Nisenbaum B, Evron E, Yerushalmi R. Everolimus plus letrozole for treatment of patients with HR(+), HER2(–) advanced breast cancer progressing on endocrine therapy: An open-label, phase II trial. *Clin Breast Cancer*. 2018;18:e197-e203 |
| 42 | Randomized phase II study of anastrozole plus tegafur-uracil as neoadjuvant therapy for ER-positive breast cancer in postmenopausal Japanese women (Neo-ACET BC) | Nakayama T, Sagara Y, Takashima T, Matsunami N, Masuda N, Miyoshi Y, Taguchi T, Aono T, Ito T, Kagimura T, et al. Randomized phase II study of anastrozole plus tegafur-uracil as neoadjuvant therapy for ER- positive breast cancer in postmenopausal japanese women (Neo-ACET BC). *Cancer Chemother Pharmacol*. 2018;81:755-762 |
| 43 | Phase III, randomized, double-blind study comparing the efficacy, safety, and immunogenicity of SB3 (trastuzumab biosimilar) and reference trastuzumab in patients treated with neoadjuvant therapy for human epidermal growth factor receptor 2-positive early breast cancer | Pivot X, Bondarenko I, Nowecki Z, Dvorkin M, Trishkina E, Ahn JH, Vinnyk Y, Im SA, Sarosiek T, Chatterjee S, et al. Phase III, randomized, double-blind study comparing the efficacy, safety, and immunogenicity of SB3 (trastuzumab biosimilar) and reference trastuzumab in patients treated with neoadjuvant therapy for human epidermal growth factor receptor 2-positive early breast cancer. *J Clin Oncol*. 2018;36:968-974 |
| 44 | Adjuvant anastrozole versus exemestane versus letrozole, upfront or after 2 years of tamoxifen, in endocrine-sensitive breast cancer (FATA-GIM3): a randomised, phase 3 trial | De Placido S, Gallo C, De Laurentiis M, Bisagni G, Arpino G, Sarobba MG, Riccardi F, Russo A, Del Mastro L, Cogoni AA, et al. Adjuvant anastrozole versus exemestane versus letrozole, upfront or after 2 years of tamoxifen, in endocrine-sensitive breast cancer (FATA-GIM3): A randomised, phase 3 trial. *Lancet Oncol*. 2018;19:474-485 |
| 45 | Addition of the PARP inhibitor veliparib plus carboplatin or carboplatin alone to standard neoadjuvant chemotherapy in triple-negative breast cancer (BrighTNess): a randomised, phase 3 trial | Loibl S, O'Shaughnessy J, Untch M, Sikov WM, Rugo HS, McKee MD, Huober J, Golshan M, von Minckwitz G, Maag D, et al. Addition of the PARP inhibitor veliparib plus carboplatin or carboplatin alone to standard neoadjuvant chemotherapy in triple-negative breast cancer (BrighTNess): A randomised, phase 3 trial*. Lancet Oncol*. 2018;19:497-509 |
| 46 | Phase III, Randomized study of dual human epidermal growth factor receptor 2 (HER2) blockade with lapatinib plus trastuzumab in combination with an aromatase inhibitor in postmenopausal women with HER2-positive, hormone receptor-positive metastatic breast cancer: ALTERNATIVE | Johnston SRD, Hegg R, Im SA, Park IH, Burdaeva O, Kurteva G, Press MF, Tjulandin S, Iwata H, Simon SD, et al. Phase III, randomized study of dual human epidermal growth factor receptor 2 (HER2) blockade with lapatinib plus trastuzumab in combination with an aromatase inhibitor in postmenopausal women with her2-positive, hormone receptor-positive metastatic breast cancer: ALTERNATIVE. *J Clin Oncol.* 2018;36:741-748 |
| 47 | Pertuzumab and trastuzumab with or without metronomic chemotherapy for older patients with HER2-positive metastatic breast cancer (EORTC 75111-10114): an open-label, randomised, phase 2 trial from the Elderly Task Force/Breast Cancer Group | Wildiers H, Tryfonidis K, Dal Lago L, Vuylsteke P, Curigliano G, Waters S, Brouwers B, Altintas S, Touati N, Cardoso F, et al. Pertuzumab and trastuzumab with or without metronomic chemotherapy for older patients with HER2-positive metastatic breast cancer (EORTC 75111-10114): An open-label, randomised, phase 2 trial from the Elderly Task Force/Breast Cancer Group. *Lancet Oncol*. 2018;19:323-336 |
| 48 | Comparing neoadjuvant nab-paclitaxel vs paclitaxel both followed by anthracycline regimens in women with ERBB2/ HER2-negative breast cancer-the evaluating treatment with neoadjuvant abraxane (ETNA) trial a randomized phase 3 clinical trial | Gianni L, Mansutti M, Anton A, Calvo L, Bisagni G, Bermejo B, Semiglazov V, Thill M, Chacon JI, Chan A, et al. Comparing neoadjuvant nab-paclitaxel vs paclitaxel both followed by anthracycline regimens in women with ERBB2/ HER2-negative breast cancer-the evaluating treatment with neoadjuvant abraxane (ETNA) trial: A randomized phase 3 clinical trial. *JAMA Oncol*. 2018;4:302-308 |
| 49 | Sequential versus simultaneous use of chemotherapy and gonadotropin-releasing hormone agonist (GnRHa) among estrogen receptor (ER)-positive premenopausal breast cancer patients: Effects on ovarian function, disease-free survival, and overall survival | Zhang Y, Ji Y, Li J, Lei L, Wu S, Zuo W, Jia X, Wang Y, Mo M, Zhang N, et al. Sequential versus simultaneous use of chemotherapy and gonadotropin-releasing hormone agonist (GnRHa) among estrogen receptor (ER)-positive premenopausal breast cancer patients: Effects on ovarian function, disease-free survival, and overall survival. *Breast Cancer Res Treat*. 2018;168:679-686 |
| 50 | Neoadjuvant letrozole for postmenopausal estrogen receptor-positive, HER2-negative breast cancer patients, a study from the Danish Breast Cancer Cooperative Group (DBCG) | Skriver SK, Laenkholm AV, Rasmussen BB, Handler J, Grundtmann B, Tvedskov TF, Christiansen P, Knoop AS, Jensen MB, Ejlertsen B. Neoadjuvant letrozole for postmenopausal estrogen receptor-positive, HER2-negative breast cancer patients, a study from the Danish Breast Cancer Cooperative Group (DBCG). *Acta Oncol.* 2018;57:31-37 |
